# Phytohormonal regulation of root exudation: mechanisms and rhizosphere function

**DOI:** 10.1080/15592324.2025.2587486

**Published:** 2025-11-22

**Authors:** Hawar Sleman Halshoy, Shwana Ahmed Braim, Jawameer R. Hama

**Affiliations:** a Department of Horticulture, College of Agricultural Engineering Sciences, University of Sulaimani, Sulaymaniyah, Kurdistan Region, Iraq; b Chemistry and Forensics Department, School of Science and Technology, Nottingham Trent University, Nottingham, UK; c Department of Agroecology, Aarhus University, Slagelse, Denmark

**Keywords:** Root exudates, plant hormones, hormonal regulation, secretion, primary metabolites, secondary metabolites, hormone signaling

## Abstract

Root exudates are pivotal mediators of plant–soil interactions, influencing nutrient acquisition, soil structure, microbial community dynamics, and plant health. These exudates comprise primary metabolites, such as sugars, amino acids, and organic acids, as well as secondary metabolites, including flavonoids, phenolics, and alkaloids, along with various enzymes and signaling molecules. Their secretion is tightly regulated by hormones, which orchestrate root development, exudate composition, and adaptive responses to environmental cues. Understanding hormones' role in the root exudation process for plant development and interaction is important; therefore, we aimed to summarize and synthesize recent findings to highlight the roles of major hormones in regulating root exudation, including auxins, cytokinins (CK), gibberellins (GA), abscisic acid (ABA), ethylene, jasmonates (JA), salicylic acid (SA), brassinosteroids (BRs), and strigolactones (SLs). The current understanding summarizes how hormone signaling pathways, crosstalk, and developmental stage transitions modulate exudate profiles, thereby shaping rhizosphere interactions. Particular attention is given to defense-related exudation under biotic and abiotic stress, nutrient mobilization, and the promotion of beneficial microbial associations. The implications of hormone-regulated exudations for sustainable agriculture are discussed, with an emphasis on strategies to enhance nutrient uptake, improve stress resilience, and reduce chemical inputs. Finally, key knowledge gaps are identified, particularly the limited integration of controlled studies with field-based complexity, and the potential for integrating emerging tools, such as hormone-responsive biosensors and metabolomics, to advance agricultural settings is discussed.

## 1. Introduction

Root exudates are a complex mixture of organic and inorganic compounds secreted by plant roots into the surrounding soil, playing a critical role in plant-soil interactions.^1^ These exudates include primary metabolites, sugars, amino acids, organic acids, phenolics, enzymes, and various secondary metabolites.^2^ The release of root exudates is essential for nutrient acquisition, altering soil structure, and mediating interactions with soil microorganisms.^3^ Root architecture influences the distribution and composition of exudates in the soil.^4^ and thus the dynamic interaction between root structure and exudation patterns determines how effectively plants exploit soil resources.^5^ While root exudates generally have positive effects, such as enhancing nutrient availability and fostering beneficial microbial communities, they can also have negative impacts.^6^ Some exudates may promote the growth of pathogenic organism^7^ or contribute to allelopathic effects that inhibit the growth of neighbouring plants.^1^ Despite these well-characterised roles, fundamental questions remain unclear regarding how plants finely regulate exudate composition in response to environmental and developmental cues.

Hormones, also known as plant hormones (hereafter hormones), are pivotal regulators of plant growth and development, influencing processes from seed germination to senescence.^8^ The relationship between root exudates and hormones is intricate and reciprocal. Hormones regulate root growth and architecture, which, in turn, affect exudation patterns.^9^ They also directly influence the composition and quantity of root exudates. Emerging evidence suggests that hormonal signalling pathways play a crucial role in modulating root exudation, affecting how plants interact with the soil environment.^10^

Despite the growing literature, significant knowledge gaps remain, particularly the molecular mechanisms by which specific hormones modulate the secretion of specific exudate compounds. It is also unclear how these regulatory pathways are integrated under multiple environmental stresses, including drought, salinity, and nutrient limitation. Moreover, the interaction between hormonal regulation and the rhizosphere microbiome has been only partially explored, with studies often yielding inconsistent results due to differences in species, experimental conditions, or analytical methods. Advances in metabolomics, transcriptomics, and rhizosphere microbiome analysis now provide tools to systematically dissect hormone-mediated exudation and its ecological consequences. A synthesis of these recent findings is urgently needed to clarify regulatory networks, identify knowledge gaps, and guide future research toward improving plant performance under real-world conditions. In this review, we aim to provide a comprehensive and mechanistic understanding of how hormones influence root exudation and, in turn, plant-soil interactions. We aimed to develop a clear narrative: first, we describe the biochemical nature and ecological significance of root exudates; next, we critically examine how different hormones regulate exudation processes; finally, we discuss the implications for sustainable agriculture, including nutrient management, stress resilience, and microbiome modulation, highlighting challenges and future directions. By explicitly connecting molecular mechanisms to ecological outcomes, this review not only addresses critical knowledge gaps but also provides a framework linking core processes to practical applications in crop production.

## 2. Overview of root exudates

Plant roots exude a considerable amount of primary and secondary metabolites into the rhizosphere, comprising 5–21% of the plant’s photosynthates.^11^ The root exudates serve multiple functions, including altering soil physical and chemical characteristics, fostering beneficial symbiotic relationships, influencing the root microbiome and soil microbial communities, and mediating interactions with other soil organisms.^12^ Secondary metabolites secreted by roots are particularly interesting, as they act as chemical signals in below-ground plant-plant interactions. Root signalling triggers a variety of adaptive responses, including root detection and recognition, defensive chemical processes, and changes in root behaviour.^13^ Belowground signalling processes have a substantial impact on plant performance above ground, particularly on flowering and reproduction,^6^ thereby influencing overall plant fitness. Understanding the composition and functions of root exudates provides insight into their pivotal role in plant growth and ecosystem functioning. However, most previous reviews have provided only descriptive accounts of root exudate components, with limited discussion of the molecular, hormonal, and ecological mechanisms that drive their synthesis and release. A more integrative approach linking the biochemical composition, hormonal regulation, and ecological outcomes of exudation is therefore essential for advancing rhizosphere science and developing sustainable crop management strategies.

### 2.1. Composition of root exudates

Root exudates comprise a diverse range of substances, including primary and secondary metabolites, enzymes, and signalling molecules. Each of these components contributes uniquely to plant physiology and interactions. Recent metabolomic and transcriptomic studies have shown that the exudate profile is not static but is dynamically regulated by both internal signals (such as hormonal status) and external factors (such as nutrient deficiency or pathogen attack). These discoveries enable a more detailed understanding of how root exudation serves as a physiological response that reflects plant–environment communication. The farmer is the main focus of this report.

#### 2.1.1. Primary metabolites

Root exudates primarily consist of sugars, amino acids, and organic acids, which are integral to a plant’s metabolic processes and crucial for its growth and development.^14^ Roots commonly release sugars, such as glucose, fructose, and sucrose, providing an essential carbon source for soil microorganisms.^15^ Amino acids such as glutamate, aspartate, and alanine facilitate nitrogen cycling in the soil and serve as signalling molecules to influence microbial behaviour.^16^ Additionally, organic acids, including citric acid, malic acid, and oxalic acid, play a crucial role in mobilising nutrients by chelating essential minerals such as iron, phosphorus, and calcium, thereby enhancing their availability for plant uptake.^17^ Recent studies have shown that phosphorus deficiency causes the release of malate and citrate through the activation of ALMT and MATE transporters^18^^,^^19^ which are regulated by hormonal signalling. These findings demonstrate that hormonal control directly links nutrient sensing to exudation processes, providing a mechanistic explanation for adaptive nutrient-seeking behaviour.

#### 2.1.2. Secondary metabolites

Root exudates contain a chemically diverse mixture of secondary metabolites, including flavonoids, phenolics, and alkaloids, derived from primary metabolic pathways but serving crucial ecological and physiological functions.^2^ The chemical structures and relative abundances of these metabolites vary widely between plant species and families, reflecting evolutionary adaptations to specific ecological niches. Within a single species, the metabolite composition can shift across growth stages, with seedlings, vegetative plants, and reproductive plants producing distinct compounds tailored to their respective developmental needs. Environmental factors, such as nutrient availability, water status, temperature, and biotic stresses, further influence root exudate profiles, enabling plants to adjust their chemical output in response to changing conditions dynamically.^6^ The synthesis and exudation of these metabolites are tightly regulated by hormones, enabling coordinated responses to both internal and external cues.^20^ For example, flavonoid biosynthesis is transcriptionally regulated by auxin and CK signalling through MYB and bHLH transcription factors,^21^ while JA and SA influence the production of phenolic antimicrobials during pathogen attack.^22^ This hormonal regulation ensures that the secretion of secondary metabolites matches defence activation,^23^ nutrient limitation,^24^ or symbiotic signalling,^25^ thereby linking molecular mechanisms to ecological functions.

#### 2.1.3. Enzymes and signalling molecules

Root exudates comprise a variety of enzymes and signalling molecules that influence soil microbial communities and plant responses to environmental factors. Enzymes such as phosphatases and proteases aid in the decomposition of organic matter, thereby releasing nutrients available for plant absorption.^26^ Signalling molecules, including hormones like auxins, CKs, and ethylene, regulate root formation and growth behaviours.^27^ Additionally, small peptides and volatile organic compounds serve as chemical signals that coordinate interactions between plants and microbes,^28^ as well as among different plants,influencing processes such as symbiosis,defence mechanisms,and competition.^25^ Recent omics-based studies have demonstrated that peptide hormones, such as the CLE and RALF families, are secreted into the rhizosphere to facilitate root–microbe communication and root flexibility.^29^^,^^30^ These signalling molecules suggest that root exudation extends beyond its nutrient functions to encompass a complex inter-kingdom communication system, with significant implications for rhizosphere manipulation and biological control strategies.^31^^,^^32^

### 2.2. Functions of root exudates

Root exudates participate in various positive and negative processes; here, we summarise the processes derived from hormones. Hormonal interactions, stress responses, and root development processes actively control the quantity and composition of exudates. These insights create opportunities for precision agriculture, including breeding strategies and the use of biostimulants, to enhance exudation, improve nutrient absorption, and enhance disease management.

#### 2.2.1. Nutrient mobilisation and uptake

One of the key roles of root exudates is assisting in the mobilisation and absorption of nutrients. Organic acids released by roots, such as citric and malic acids, bind to soil mineral ions, thereby increasing their solubility and making them more readily available for plant uptake. This is particularly important for phosphorus absorption, which is often restricted in soils due to its low solubility.^17^ Additionally, exudates can affect the pH of the rhizosphere, increasing the solubility of certain nutrients.^33^ For instance, the release of hydrogen ions can lower soil pH, thereby improving the availability of iron and manganese from soil particles. Experimental work has demonstrated that auxin and ethylene enhance the expression of malate transporters (AtALMT1) in response to phosphorus deficiency, thereby increasing root exudation of organic acids and improving nutrient uptake efficiency.^34^^,^^35^ This hormone-mediated regulation highlights the adaptive connection between nutrient signalling and exudation patterns, offering practical targets for developing nutrient-efficient crop varieties.

#### 2.2.2. Attraction of beneficial microbes

Root exudates play a crucial role in attracting beneficial soil microbes, such as Arbuscular Mycorrhizal Fungi (AMF) and Rhizobia.^36^ Flavonoids found in legume root exudates serve as signalling molecules, activating the expression of nodulation (nod) genes in Rhizobia and initiating the development of nitrogen–fixing root nodules.^37^ Similarly, SLs, a type of hormone found in root exudates, promote the germination and hyphal branching of AMF, facilitating the establishment of mycorrhizal symbiosis.^38^ These mutually beneficial relationships enhance nutrient uptake, particularly for nitrogen and phosphorus. At the molecular level, the synthesis of SLs is controlled by the auxin and CK pathways through the MAX and D27 gene families, indicating a direct hormonal influence on symbiotic recruitment.^39–41^ Modifying these signalling pathways could enhance root–microbe relationships and decrease reliance on synthetic fertilisers.^42^

#### 2.2.3. Defence against pathogens and pests

Root exudates contribute to plant defence against soil-borne pathogens and pests by secreting antimicrobial compounds and modulating microbial communities.^43^ Root exudates, which contain phenolic compounds and alkaloids, exhibit antimicrobial effects that hinder the growth of harmful bacteria and fungi.^1^ Furthermore, some exudates promote the growth of beneficial microbes, such as biocontrol agents, which combat pathogens through mechanisms including competition, antibiosis, or induced systemic resistance.^44^ For instance, releasing certain sugars and amino acids can enhance the proliferation of plant growth-promoting rhizobacteria. JA and SA signalling pathways are crucial regulators of defence-related exudation, triggering the secretion of antimicrobial phenolics and terpenoids.^45^^,^^46^ These are the mechanistic bases for designing elicitor-based biostimulants that enhance rhizosphere resistance to pathogens through targeted hormonal priming.

#### 2.2.4. Allelopathic interactions with neighbouring plants

Allelopathy is the chemical interaction between plants, mediated by the release of bioactive compounds into the rhizosphere, which act as germination or growth inhibitors.^47^ Root exudates contain allelochemicals, such as phenolics and alkaloids, which can inhibit the germination and growth of nearby plants, thereby reducing competition for resources.^48^ This phenomenon is commonly observed in agricultural systems, where certain crops release allelopathic substances that suppress weed growth.^49^ For instance, the compound sorgoleone, released by sorghum roots, has been shown to hinder the growth of competing plant species.^50^ Further, benzoxazinoids function as key allelochemicals in cereals, mediating allelopathic interactions by being exuded from roots and subsequently degrading into phytotoxic compounds such as benzoxazolinones, which suppress the germination and growth of competing plant species.^51^ Allelopathic relationships can influence plant community structure and biodiversity in natural ecosystems.^52^ Recent research indicates that the ABA^53^ and JA^54^ signalling pathways regulate the production and secretion of allelochemicals in response to drought or nutrient stress. Understanding these hormonal controls offers promising opportunities to breed or engineer crops with enhanced natural weed suppression, thereby reducing dependence on herbicides.^55^

## 3. Key hormones regulating root exudation

**Figure 1.**
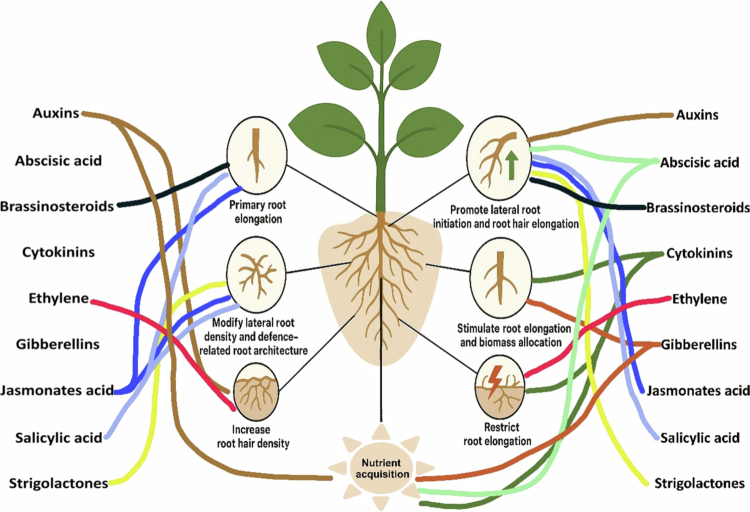
Hormones are involved in plant physiological processes during root exudation.

The main groups of hormones involved in regulating root exudation processes are auxins, CKs, GAs, ABA, ethylene, JA, SA, BRs, and SLs. The chemical structures of the major hormones involved in root exudation are summarised in Table 1. Furthermore, the plant physiological processes regulated by these hormones during root exudation are illustrated in Figure 1. Although the functions of these hormones are well understood, recent advances in transcriptomics, metabolomics, and imaging technologies have shown that their regulatory effects are coordinated across space and time through complex signalling networks. Understanding these hormonal interactions offers new opportunities to regulate root exudation, enhancing nutrient use, stress tolerance, and rhizosphere management.

**Table 1.** The major roles of hormones in regulating root exudation, associated metabolites, and ecological/agricultural outcomes.

Hormone	Effect on root architecture	Major exudates influenced	Environmental/stress context	Ecological or agricultural implications	Reference
Auxins	Promote lateral root initiation and root hair elongation	Organic acids (malate, citrate), flavonoids, and amino acids	Nutrient deficiency (e.g., phosphorus, iron)	Enhance rhizosphere acidification, mobilise soil nutrients, and recruit plant growth-promoting rhizobacteria	[56,57]
Cytokinins (CK)	Inhibit primary root elongation; balance shoot–root growth	Sugars and phenolics	High nitrate and nutrient-rich soils	Alter the rhizosphere microbial community, regulate carbon allocation, and nitrogen assimilation	[58]
Gibberellins (GA)	Stimulate root elongation and biomass allocation	Carbohydrates and secondary metabolites	Plant growth and recovery after abiotic stress	Enhance root–shoot signalling and support Arbuscular Mycorrhizal Fungal symbiosis	[59,60]
Abscisic acid	Restrict root elongation and enhance root hair density under stress	Proline, osmolytes, and organic acids	Drought, salinity, and osmotic stress	Improve water-use efficiency, modulate stress-induced microbial associations	[61,62]
Ethylene	Increase root hair density; inhibit primary root elongation	Phenolics and volatile organic compounds	Mechanical stress and pathogen attack	Activate plant defence and stimulate rhizobacteria-mediated growth promotion	[2,63]
Jasmonates (JA) and salicylic acid (SA)	Modify lateral root density and defence-related root architecture	Terpenoids, alkaloids, and phenolics	Pathogen infection, herbivory, and biotic stress	Enhance immunity, recruit beneficial microbes, and induce allelopathic interactions	[64]
Brassinosteroids (BRs) and strigolactones (SLs)	BRs promote root elongation and cell division, while SLs suppress lateral roots and enhance mycorrhizal symbiosis.	SLs, coumarins, and flavonoids	Phosphate deficiency and symbiotic interactions	Improve phosphorus acquisition, mediate parasitic weed interactions, and enhance beneficial mycorrhizal recruitment	[41,65]

### 3.1. Auxins

Auxins are pivotal hormones that significantly influence root exudation by altering root architecture and affecting rhizosphere dynamics.^56^^,^^66^ Auxins are synthesised in the shoot apical meristem and transported to the roots, which play a central role in root elongation, lateral root formation, and overall root system development, directly impacting the quantity and composition of root exudates.^67^ Auxin-induced changes enhancing exudation and microbial recruitment.^68^ Auxins regulate gene expression related to exudate secretion, facilitating the targeted release of compounds that attract beneficial microbes while potentially deterring pathogens.^69^^,^^70^ Environmental factors, such as nutrient availability and stress, can modulate auxin levels, resulting in adaptive exudation patterns that enhance plant resilience.^26^ A deeper understanding of auxin's role in root exudation can leverage plant-microbe interactions to boost crop yields and soil health.

This spatial regulation is achieved through polar auxin transporters, such as the PIN-FORMED (PIN) and AUX1/LAX families, which create local auxin gradients that define “exudation hotspots” along the root.^71^^,^^72^ Disruption of these transporters in *Arabidopsis* mutants alters both the composition and total carbon flux of root exudates, underscoring the mechanistic link between auxin distribution and metabolite secretion.^73^ At the molecular level, auxin signalling through auxin response factor (ARF)–indole−3-acetic acid (IAA) modules modulates the transcription of genes involved in transporter activity and secondary metabolism.^74^^,^^75^ For instance, auxin-induced expression of malate and citrate efflux transporters (AtALMT1, MATE) under phosphorus deficiency enhances organic acid exudation, thereby mobilising insoluble phosphorus.^19^ Similarly, auxin signalling stimulates flavonoid biosynthetic genes through MYB–bHLH transcription factor complexes, influencing the secretion of flavonoids that act as chemoattractants for beneficial rhizobacteria. Manipulating auxin biosynthesis or signalling, through genetic engineering, biostimulants, or microbe-assisted strategies, can enhance root exudation profiles that favour nutrient acquisition and beneficial microbial recruitment. This offers promising potential for developing low-input cropping systems that improve soil health and plant productivity under nutrient or water limitations. Collectively, these insights demonstrate that auxin functions as a central integrator connecting molecular signalling, root physiology, and rhizosphere ecology. A deeper mechanistic understanding of auxin-regulated exudation will enable the design of targeted interventions to optimise root–microbe–soil interactions and enhance crop performance.

### 3.2. Cytokinins

Cytokinins (CK) are adenine-derived hormones that primarily regulate cell division and differentiation, influencing various aspects of root system architecture and rhizosphere interactions. CKs regulate the exudation of organic acids and amino acids, thereby enhancing nutrient absorption, particularly in nutrient-deficient soils.^76^ Beyond their classical role in shoot–root signalling, CKs have recently been recognised as key modulators of root exudation, affecting both the composition and signalling functions of rhizosphere exudates. They also foster beneficial relationships with AMF and nitrogen-fixing bacteria, promoting robust plant growth and development. Interestingly, CKs interact with auxins to regulate root exudation, thereby balancing root growth and function, which, in turn, influences the types and amounts of exudates released.^77^ CKs regulate the balance between root and shoot growth by modulating auxin transporter expression and root meristem activity. At the root level, CK-mediated regulation of transporter genes, such as SWEET sucrose effluxers and ABC transporters, influences the carbon allocation to roots and thus the availability of substrates for exudation. In *Arabidopsis*, exogenous application of zeatin or trans-zeatin riboside has been shown to decrease the exudation of organic acids but increase the release of amino acids and phenolic compounds. This hormonal shift suggests that CKs fine-tune the quality rather than the total quantity of exudates, likely optimising rhizosphere signalling under specific environmental contexts. CKs signalling through the CRE1/AHK–ARR pathway influences the expression of amino acid and sugar efflux transporters, thereby adjusting nutrient-related exudation.^78^^,^^79^ Additionally, CK-driven repression of auxin transport can alter root-zone exudation profiles, thereby affecting microbial assembly in the rhizosphere. This hormonal balance presents a potential target for enhancing the effectiveness of microbial inoculants in sustainable agriculture. Modulating CK signalling, either through exogenous application, genetic manipulation of IPT/CKX genes, or microbial inoculants that produce CKs, offers potential to enhance beneficial plant–microbe interactions and stress resilience. By adjusting the metabolic profile of root exudates, CK-based interventions could help optimise nutrient use efficiency and reduce dependence on chemical fertilisers in sustainable cropping systems.

### 3.3. Gibberellins

Gibberellins (GA) regulate root exudation by influencing both the quantity and composition of exudates across different growth stages.^80^ These hormones promote cell elongation and growth, affecting root development.^81^ During early growth stages, GAs enhance root elongation, increasing the volume of exudates released into the rhizosphere. As plants mature, GAs target the release of specific metabolites essential for physiological function.^59^ GAs modulate the exudation of organic acids, sugars, and amino acids, enhancing nutrient uptake and shaping the rhizosphere microbiome, and regulating the release of secondary metabolites, influencing soil microbe populations and nutrient cycling.^82^ Molecularly, GA signalling via DELLA proteins interacts with auxin and CK pathways to regulate exudation patterns.^83^ In rice, GA-driven degradation of DELLAs promotes root elongation and the release of phenolic acids that encourage beneficial bacterial colonisation.^84^ These regulatory insights could support the use of GA analogues to boost root–microbe cooperation in nutrient-deficient soils.^85^

### 3.4. Abscisic acid

Abscisic acid is traditionally recognised as a “stress hormone” that regulates plant responses to drought, salinity, and other environmental constraints.^62^ Recent findings indicate that ABA also plays a central role in shaping root exudation patterns under stress, coordinating carbon fluxes, transporter activity, and microbial communication.^86^ Abscisic acid regulates osmotic balance in root cells, enhances water retention, and influences the composition of exudates.^87^ It regulates the release of osmoprotectants, such as proline, sugars, and organic acids, which maintain cellular turgor and protect root cells from dehydration.^61^ ABA signalling through PYR/PYL receptors activates SNF1-related protein kinase2 (SnRK2) cascades, which, in turn, regulate the expression of transporters such as the MATE, ALMT, and NRT families. These transporters mediate the release of organic acids, amino acids, and compatible solutes, such as proline and betaine, which can alleviate osmotic stress and modulate microbial activity in the rhizosphere. At the molecular level, ABA detection by PYR/PYL/RCAR receptors activates SnRK2 kinases, which then phosphorylate transcription factors like AREB/ABF. These factors regulate genes involved in transporter activity and metabolite secretion.^88^^,^^89^ ABA-induced root exudation of compatible solutes, such as trehalose, has been shown to support drought-resistant microbial communities,^90^ highlighting potential strategies to enhance rhizosphere drought resilience through hormonal priming.

Experimental evidence supports this regulatory connection. For instance, ABA-deficient mutants (*aba1*, *aba2*) in *Arabidopsis* show reduced exudation of sugars and organic acids under drought stress, correlating with impaired recruitment of drought-protective rhizobacteria. Similarly, exogenous ABA application in maize increased root secretion of malate and citrate, thereby enhancing rhizosphere phosphorus mobilisation under phosphorus-limited conditions. These findings suggest that ABA acts as a metabolic switch, reallocating resources from growth to adaptive rhizosphere functions.

### 3.5. Ethylene

Ethylene, a gaseous hormone, plays a crucial role in regulating root exudation, especially under biotic and abiotic stress conditions.^91^ Ethylene influences the types and quantities of root exudates. Under abiotic stresses, ethylene levels increase, altering exudation patterns.^92^ This results in a greater release of amino acids, organic acids, and secondary metabolites. In biotic stress situations, such as pathogen attacks, ethylene triggers the release of defensive compounds, including phytoalexins and antimicrobial peptides, inhibiting pathogen growth.^93^ Ethylene also regulates signalling molecules that attract beneficial microbes, enhancing plant defences.^94^ Beyond stress responses, ethylene influences normal root development, promoting root hair and lateral root formation, increasing root surface area, and exudation potential.^2^ Ethylene works synergistically with other hormones, such as auxins and abscisic acid, to coordinate root development and exudation. Experimental studies have shown that ethylene-insensitive *Arabidopsis* mutants (ein2, etr1) exhibit altered exudate composition and reduced colonisation by rhizobacteria, confirming the hormone’s role in microbial recruitment.^95^^,^^96^ Additionally, ethylene-triggered production of phenolic and amino acid exudations, which helps maintain microbial balance in the rhizosphere under stress, indicates that ethylene modulation could be a promising strategy for biocontrol in crop protection.

### 3.6. Jasmonates and salicylic acid

Jasmonates (JA) and salicylic acid (SA) regulate defence-related root exudation, which is crucial for plant responses to biotic stress and rhizosphere interactions. JAs are primarily associated with defence against necrotrophic pathogens and herbivorous insects,^97^ while SA plays a significant role in resisting biotrophic pathogens.^98^ Hormones influence the quantity and composition of exudate, repelling pathogens or attracting beneficial microbes. JAs promote the release of secondary metabolites, such as phenolics, flavonoids, and terpenoids,^99^ which possess antimicrobial properties and deter herbivores. SA encourages the secretion of defence compounds,such as pathogenesis-related proteins and phenolic acids,enhancing systemic acquired resistance against pathogens.^100^ The complex crosstalk between JA and SA fine-tunes defence mechanisms and exudate profiles. This interaction can be antagonistic or synergistic, depending on the type of stress and environmental conditions. For instance, SA signalling can suppress JA responses and vice versa, optimising plant resources for specific threats.^64^ Recent transcriptomic analyses revealed that JA-activated MYC2 transcription factors upregulate terpene synthase genes,^101^ increasing root exudation of antimicrobial volatiles that suppress soilborne fungi.^102^ Conversely, SA signalling through NPR1 enhances the secretion of benzoic acid derivatives that promote beneficial microbial recruitment^103^^,^^104^ Understanding these molecular interactions provides a foundation for deploying hormone elicitors to strengthen rhizosphere immunity in integrated pest management.

### 3.7. Brassinosteroids and strigolactones

Brassinosteroids (BRs) promote cell elongation and division, which enhances root elongation and lateral root formation, increasing root surface area and exudation potential.^105^ BRs also modulate the secretion of primary and secondary metabolites, which are vital for nutrient acquisition and microbial interactions. Strigolactones (SLs) inhibit shoot branching while regulating root development.^106^ They act as signalling molecules in the rhizosphere, facilitating symbiotic associations with AMF. The interaction between BRs and SLs dynamically regulates root architecture and exudate profiles, optimising plant adaptation to environmental conditions. Their combined effects highlight the complexities of hormonal regulation in shaping root function and rhizosphere dynamics.^41^^,^^65^ BRs regulate flavonoid and phenolic exudations through the transcription factors BZR1/BES1, supporting microbial colonisation and stress relief.^107^ These molecular insights underscore the practical potential of hormonal cross-regulation to create biofertilizer-compatible cropping systems. Additionally, SL biosynthesis, involving D27, CCD7, CCD8, and MAX1 genes, is precisely controlled by auxin and nutrient levels to enable context-specific exudation that attracts AMF.^108^^,^^109^

## 4. Mechanisms of hormonal control on root exudation

Hormonal regulation is essential for controlling both the quantity and composition of root exudates, with hormones orchestrating complex signalling pathways. Root exudation is a highly dynamic process, regulated across space and time, with hormone-dependent mechanisms varying among plant species, root zones, and environmental conditions. Below, we summarise the major mechanisms by which hormones influence signalling, crosstalk, and adaptive responses in the rhizosphere.

### 4.1. Hormone signalling pathways

Hormonal signalling pathways regulate root exudation through interconnected networks of perception, transduction, and gene expression.^110^ For instance, in *Arabidopsis*, auxin perception by TIR1/AFB receptors at root tips activates ARF transcription factors, which, in turn, regulate genes encoding malate and citrate transporters, such as *ALMT1* and *MATE*.^111^ This regulation is spatially localised, as auxin maxima occur at root apices and young lateral roots, where the exudation of organic acids supports nutrient mobilisation and microbial colonisation. Similarly, in rice and maize, auxin signalling promotes the exudation of flavonoids and amino acids from root hairs, shaping the rhizosphere microbiome. CK signalling, mediated by histidine kinase receptors (CRE1/AHK family), often acts in opposition to auxin signalling. High CK levels under nutrient sufficiency repress auxin transporters (*AUX1* and *PINs*), reducing organic acid exudation from elongated zones.^112^ Abscisic acid (ABA) signalling through PYR/PYL–SnRK2–ABF modules is activated under drought or osmotic stress, inducing transcriptional upregulation of SWEET sugar transporters and genes linked to osmoprotectant secretion, including proline and trehalose.^113^ Ethylene, sensed by EIN2/EIL1, promotes phenolic and amino acid exudation in root hair zones, facilitating microbial colonisation and stress resilience. Transporters, such as ABC transporters, PIN proteins, and aquaporins, coordinate these hormonal signals with spatial control, enabling localised exudate secretion at root tips, root hairs, or lateral roots, depending on the plant’s developmental stage and environmental conditions.^26^ Experimental studies across species have shown that the TIR1/AFB–ARF pathway controls flavonoid exudation in root hairs, while malate exudation in *Arabidopsis* is mainly regulated at the root apex, demonstrating species- and tissue-specific responses.^114^

### 4.2. Crosstalk between hormonal pathways

Root exudation is not governed by individual hormones acting in isolation but rather by synergistic and antagonistic crosstalk among multiple signalling networks. Auxins and CKs often function synergistically to regulate nutrient uptake and carbon allocation, thereby influencing the composition and quantity of root exudates.^115^ Furthermore, under nitrogen limitation, their joint action enhances the exudation of amino acids and sugars, thereby promoting beneficial microbial colonisation. In contrast, abscisic acid typically antagonises GAs and ethylene during stress, prioritising the secretion of osmoprotective compounds. Crosstalk between JA and ethylene enhances the release of secondary metabolites and flavonoids during herbivory or pathogen attack, as observed in tomato and *Arabidopsis*.^116^ Meanwhile, JA and SA exhibit antagonism, with SA promoting the exudation of phenolics that deter biotrophic pathogens, while JA enhances the release of defence-related terpenoids against necrotrophs.^117^

These interactions converge on shared transcriptional hubs, such as MYC2, ABF, and NAC proteins, which integrate hormonal cues to fine-tune the expression of transporter genes that control amino acid and sugar efflux.^118^^,^^119^ Importantly, this crosstalk is context-dependent and species-specific: for example, *Arabidopsis* and maize exhibit different patterns of flavonoid and phenolic exudation in response to auxin–CK and JA-ethylene interactions.

### 4.3. Environmental and developmental modulation

Environmental conditions and developmental stage strongly influence the hormonal regulation of root exudation.^120^ Nutrient availability strongly modulates hormone-driven exudation: phosphorus deficiency increases root auxin levels, stimulating lateral root growth and citrate/malate exudation in *Arabidopsis* and wheat roots.^121^ Similarly, nutrient stress elevates ethylene production, enhancing exudation of micronutrient-mobilising compounds such as iron chelators.^122^ CKs suppress exudation when nutrients are sufficient, prioritising shoot growth, whereas abscisic acid predominates under drought, inducing osmoprotective exudates such as proline and trehalose.^62^^,^^90^

Root exudation patterns shift as the plant matures. During seedling establishment, auxin and CK jointly promote the secretion of low-molecular-weight sugars and amino acids from root tips and hairs, which support beneficial microbial colonisation.^58^ During the vegetative stage, GAs and BRs enhance the release of secondary metabolites from elongation zones, improving nutrient acquisition and rhizosphere signalling.^123^ At reproductive stages, elevated ABA and JA levels drive the exudation of phenolics, terpenoids, and other defence-related metabolites, thereby strengthening stress tolerance and altering root–microbe interactions.^124^ Overall, hormonal control of root exudation is context-dependent and species-specific, requiring spatially resolved metabolomics and transcriptomic analyses to uncover the dynamic interplay between hormonal signalling, development, and the environment.

## 5. Implications for agriculture and ecosystem management

The regulation of root exudation by hormones provides clear chances to enhance crop productivity, soil health, and ecosystem resilience. However, applying these mechanisms from laboratory research to real-world fields demands species-specific strategies, consideration of environmental differences, and awareness of current technological limits.

### 5.1. Enhancing nutrient uptake and soil fertility

Manipulating hormones presents a promising approach to enhancing nutrient uptake, soil fertility, and overall agricultural sustainability. Hormones regulate root architecture and exudate secretion, directly affecting nutrient availability and beneficial microbial interactions.^125^ For instance, auxin-driven lateral root development in maize and wheat improves phosphorus and nitrogen absorption under low-fertility conditions.^121^^,^^126^ Similarly, root exudation of organic acids, for example, in rice^127^^,^^128^ and soybean^129^ helps release bound phosphorus in acidic soils, increasing nutrient accessibility. These exudates also support beneficial microbes, such as nitrogen-fixing bacteria and Arbuscular Mycorrhizal Fungi (AMF), thereby improving soil structure and microbial diversity. Field studies show that combining AMF inoculation with auxin treatments can increase phosphorus uptake and boost microbial biomass.^130^ Hormonal regulation thus offers a sustainable method to reduce reliance on chemical fertilisers while lowering nutrient runoff and soil degradation. Nonetheless, variable responses among cultivars and challenges in managing hormone application under field conditions emphasise the need for integrated soil–plant management strategies.

### 5.2. Promoting beneficial plant-microbe interactions

Hormones play a crucial role in facilitating beneficial interactions with mycorrhizal fungi^85^ and nitrogen-fixing bacteria,^131^ thereby enhancing nutrient uptake,promoting plant health,and increasing stress resilience. Key hormones regulate symbiotic development from initial signalling to sustained mutualism. SLs stimulate the germination of AMF spores and hyphal branching,thereby enhancing root colonisation and phosphorus uptake.^132^ In nitrogen-fixing symbioses, CKs drive nodule formation,^133^ auxins aid root cell differentiation,^134^ and ethylene balances nodule development.^135^ Enhancing these pathways via external applications, genetic engineering, or selective breeding can optimise AMF colonisation and nodulation efficiency. This reduces reliance on synthetic fertilisers, mitigates environmental impacts, and promotes soil biodiversity, advancing sustainable agriculture and ecosystem resilience. However, practical constraints still exist: external hormone applications can be expensive, environmentally sensitive, and crop-specific, and genetic modifications encounter regulatory challenges. Therefore, site- and crop-specific strategies that combine microbial inoculants with hormone-responsive varieties are crucial for real-world implementation.

### 5.3. Stress tolerance and sustainable crop production

Hormones regulate root exudation, which affects stress tolerance and sustainable crop production. Under stress conditions such as drought, salinity, and nutrient deficiencies, hormonal signalling adjusts exudate composition to enhance resilience. Abscisic acid regulates the release of osmoprotectants, thereby reducing oxidative damage,^136^ while ethylene and JA promote the release of defence-related exudates.^137^ SLs enhance AMF colonisation, facilitating improved nutrient absorption,^138^ while auxins stimulate root growth,thereby expanding microbial interactions.^139^ Fine-tuning these hormonal pathways through external application or genetic modification enhances plant defences, thereby reducing the need for chemical inputs. Field studies show that combining hormonal treatments with AMF inoculation in maize under drought conditions increases yields compared with untreated controls.^140^ SL-enhanced AMF colonisation also increases phosphorus uptake during nutrient stress.^141^ Challenges for practical use include variable responses across different soil types, microbial communities, and crop genotypes. Additionally, external hormone applications or genetic modifications need to be carefully timed and dosed to prevent unintended ecological effects. Therefore, integrated management strategies that combine hormone manipulation, microbial inoculation, and adaptive breeding are recommended for scalable and sustainable implementation. Thus, hormone-regulated root exudation presents significant opportunities to enhance crop nutrition, resilience, and soil health. Turning these findings into practical applications requires species-specific methods, field-trial evidence, and awareness of technological and environmental constraints.

## 6. Outlook and future perspectives

Most current studies on root exudation have been conducted under controlled growth conditions, which, while useful for mechanistic insights, often fail to capture the full complexity of natural soils. Field studies on crops such as maize,^142^ switchgrass,^143^ and wheat,^144^ rice^145^ have shown that root exudation profiles observed in controlled environments differ significantly from those in heterogeneous soils, with microbial community composition and nutrient levels strongly influencing the amount and quality of exudates.^12^ In real-world environments, root exudation is influenced not only by intrinsic hormonal signalling but also by complex interactions with soil type, nutrient availability, microbial communities, and abiotic stresses such as drought, salinity, and heat. For example, drought-stressed maize exhibits increased abscisic acid-induced exudation of osmoprotectants, while phosphorus-limited soybean increases citrate exudation to mobilise *P*, emphasising species- and context-specific hormonal responses. The crosstalk between hormones under these conditions is only partly understood, including antagonistic interactions between ethylene and abscisic acid during drought stress, and the synergistic role of JA and SA in defence. To understand these interactions in real-world settings, combined approaches that connect field experiments with mechanistic studies at the cellular and molecular levels are necessary.

Emerging technologies are creating new opportunities to bridge the current knowledge gap between mechanistic insights on hormone-regulated root exudation and field-level applications. CRISPR–Cas9 genome editing has been employed to target key auxin transporters and regulatory genes in *Arabidopsis* and maize, revealing their role in shaping root exudate composition and influencing rhizosphere microbial communities.^146^ For example, knocking out specific PIN-family auxin transporters in *Arabidopsis* led to localised changes in malate and citrate exudation at the root apex, which subsequently altered microbial recruitment in the rhizosphere.^147^ In maize, CRISPR-mediated modification of auxin transport pathways increased lateral root density, leading to greater exudation of amino acids and sugars and promoting colonisation by beneficial nitrogen-fixing bacteria.^148^ While these studies demonstrate the potential of genome editing for optimising root exudation, challenges remain for field implementation, including crop-specific responses, off-target effects, and regulatory constraints on genetically modified crops.

Biosensors, particularly FRET-based hormone reporters, have enabled real-time monitoring of hormonal dynamics in root tissues, providing spatial and temporal resolution of auxin, CK, and abscisic acid fluctuations. For instance, auxin sensors in *Arabidopsis* root tips revealed that fluctuations in hormone levels at root hairs and lateral root initiation sites correlate with the targeted exudation of flavonoids and organic acids.^149^^,^^150^ Similarly, CK-responsive biosensors in wheat roots demonstrated that local hormone gradients are linked to sugar and amino acid release under nutrient-limited conditions. By combining biosensor technology with microfluidic growth platforms, researchers can visualise and quantify exudate responses to environmental cues in real time, bridging the gap between laboratory mechanistic studies and applied crop management. However, scaling these approaches to field conditions remains challenging due to sensor sensitivity, environmental variability, and cost constraints.

Precision agriculture systems that integrate soil sensors, automated irrigation, and nutrient-delivery platforms offer practical solutions for applying hormone- and exudation-informed management strategies. Field studies in wheat and maize have demonstrated that site-specific nutrient delivery, guided by soil sensors, can increase nutrient-use efficiency while minimising fertiliser runoff.^151^ When combined with knowledge of hormone-regulated exudation, these platforms allow for crop-specific interventions, such as timing fertiliser application to coincide with peaks in auxin- or CK-mediated root activity, thereby enhancing nutrient mobilisation and microbial recruitment. For instance, targeted application of phosphorus fertilisers in maize plots timed with auxin-induced lateral root development increased phosphorus uptake and AMF colonisation compared with conventional uniform fertilisation.^110^ Limitations of this approach include high initial costs, variable responses across soil types, and the need to calibrate sensors and algorithms for each crop.

Collectively, these advanced technologies provide complementary tools for dissecting, monitoring, and manipulating hormone-regulated root exudation. Integrating mechanistic studies, real-time monitoring, and field-level interventions offers a pathway to practical, crop-specific strategies to improve nutrient uptake, enhance soil health, and promote sustainable agriculture. Future work should focus on cross-validating laboratory findings in multi-species field trials, developing scalable sensor platforms, and establishing regulatory frameworks for the responsible deployment of gene-edited crops.

The application of this knowledge has strong potential for sustainable agricultural innovation. By combining molecular tools with applied agronomic practices, crops can be optimised for better nutrient uptake, enhanced water-use efficiency, increased tolerance to abiotic stress, and more beneficial plant–microbe relationships. For instance, in field trials with rice, exogenous SL analogues improved AMF colonisation and phosphorus uptake, decreasing the need for synthetic fertilisers.^130^ However, practical challenges include crop-specific responses, variation in microbial communities, and regulatory and cost constraints related to gene editing or synthetic hormone use.

To advance the practical implementation of hormone-regulated root exudation in sustainable agriculture, we propose a stepwise, integrative research framework:

1. Conduct targeted experiments in growth chambers or hydroponic systems to dissect hormone-regulated exudate pathways and identify specific metabolites. For instance, CRISPR–Cas9 editing of PIN-family auxin transporters in *Arabidopsis* and maize has revealed their role in localised exudation of malate, citrate, and amino acids, which in turn affects microbial recruitment. Controlled studies allow precise manipulation of hormonal levels, environmental variables, and root developmental zones to uncover the molecular and spatial mechanisms driving exudation.
2. Validate laboratory findings under diverse soil types, climates, and crop cultivars. For instance, field trials in maize and rice have demonstrated that auxin- and SL-mediated root exudation enhances phosphorus uptake and AMF colonisation, improving nutrient-use efficiency. These experiments help identify species- and genotype-specific responses, ensuring that mechanistic insights translate into real-world productivity gains.
3. Combine transcriptomics, metabolomics, and microbiome profiling to link hormonal signalling, exudate composition, and microbial recruitment. Integrating hormone-responsive transcriptomic data with rhizosphere metabolomics has shown that auxin-regulated flavonoid exudation correlates with increased colonisation by nitrogen-fixing bacteria in soybean. Multi-omics approaches provide comprehensive maps of plant–microbe–environment interactions, identifying key metabolites and regulatory nodes for targeted interventions.
4. Apply knowledge of hormone-regulated exudation in the field using precision agriculture platforms, including soil sensors, automated irrigation, and nutrient delivery systems. By timing fertiliser or hormone-based interventions to coincide with peaks in auxin- or CK-mediated root activity, nutrient uptake efficiency and microbial recruitment can be maximised. Such site-specific management minimises environmental impacts and enhances the effectiveness of hormone-informed strategies.
5. Combine laboratory mechanistic data and field trial results to inform crop improvement strategies. This may include breeding for hormone-responsive cultivars or applying CRISPR-based modifications to enhance root exudation traits linked to nutrient acquisition, microbial recruitment, and stress. Iterative feedback allows refinement of strategies, ensuring that field-ready crops are resilient, high-performing, and environmentally sustainable.

By adopting these integrative, stepwise methods, research can move beyond theory to develop practical, field-ready strategies that harness hormone-regulated root exudation. This approach links mechanistic understanding, advanced monitoring, and applied agronomy, enabling crops to achieve higher yields, improved nutrient-use efficiency, and enhanced soil and ecosystem health while maintaining environmental sustainability.

## Data Availability

Data are available from the corresponding author upon reasonable request.
